# Pharmacodynamics and pharmacokinetics of a new type of recombinant insulin Lisargine injection

**DOI:** 10.1186/s12906-020-03110-3

**Published:** 2020-11-09

**Authors:** Jiangjie Lu, Yong Zeng, Xiulin Yi, Hongmei Zhang, Lin Zhu, Lixin Jiang, Jing Li, Wei Zhou, Hong Zhu, Aijun Xiong

**Affiliations:** 1 Hefei Tianmai Biotechnology Development Co,. Ltd., Hefei, 230601 Anhui Province China; 2 TIPR Drug Assessment Co., Ltd, Tianjin, 300452 China; 3 WuXi AppTec Co., Ltd (Headquarters), Shanghai, 200131 China; 4 WuXi AppTec Co., Ltd (Suzhou), Suzhou, 215104 Jiangsu Province China

**Keywords:** Pharmacodynamic, Pharmacokinetic, Recombinant insulin Lisargine injection, Lantus

## Abstract

**Background:**

Recombinant insulin Lisargine is a new type of insulin. In this study, we aimed to compare its pharmacodynamic (PD) and pharmacokinetic (PK) with Lantus.

**Methods:**

The PD test was performed by exploring the effect of single administration on blood glucose of normal rats and STZ-induced diabetic rats, and the effect of multiple administrations on blood glucose of STZ-induced diabetic rats. Further PD tests include receptor affinity test, receptor autophosphorylation test and adipocyte glucose uptake test. Four IU and 8 IU per dog Lisargine was used for PK test, insulin was measured and area under curve (AUC) was calculated.

**Results:**

With single injection, Lisargine 1.5 IU/kg had significant hypoglycemic effects at 1 and 2 h, similar to that of Lantus. Lisargine 5 IU/kg and 10 IU/kg lowered the blood glucose of STZ-induced diabetic rats at 1, 2, 4 & 6 h significantly. With multiple injections, Lantus lowered blood glucose at 2, 4 & 6 h, Lisargine 2.5 IU/kg, 5 IU/kg, and 10 IU/kg lowered blood glucose at 2 & 4 h significantly, compared with vehicle. There was no difference for receptor affinity test, receptor autophosphorylation test and adipocyte glucose uptake test between Lisargine and Lantus. The PK of Lisargine and Lantus of healthy Beagle dogs was very similar.

**Conclusions:**

This animal study demonstrated that PK and PD of Lisargine and Lantus were similar, suggesting the bioequivalence of these products.

## Background

Diabetes Mellitus is one of the fast growing non-communicable diseases and a threat to global public health [1]. Many complications such as diabetic nephropathy, retinopathy, neuropathy, delayed wound healing, heart attack, peripheral vascular disturbances and diabetic ketoacidosis could happen, jeopardizing the quality of life of the patients and adding great burden to the family and community [2–4]. One major pathogenesis is its prevailing condition hyperglycemia. Thus, effective and reliable hypoglycemic agent is very important.

One widely used hypoglycemic agent is insulin and its analogs [5–9]. Lantus (Insulin glargine) was approved by the FDA on April 20, 2000. It is a long-acting, human insulin analogue that has been specifically designed to overcome the deficiencies of traditionally available ‘intermediate-acting’ insulin used for basal insulin supplementation [10].

Lysine lowers blood glucose. At home and abroad, no lysine insulin product is currently available in the market. With recombinant technology, we produced insulin Lisargine injection, a new type of insulin with lysine. It consists of 53 amino acid residues. Its chemical name is ^21^A-Gly-^30^Ba-L-Lys-^30^Bb-L-Arg-human insulin. In this study, by using Lantus as a positive control, we explored the pharmacodynamic (PD) and pharmacokinetic (PK) of recombinant insulin Lisargine injection.

## Methods

### Materials

Recombinant insulin Lisargine injection (Lot No. M201605003) (Lisargine) was obtained from Hefei Tianmai Biotechnology Development Co., Ltd. The recombinant human-derived insulin (SLBL1965V/12643) was from Sigma (S-Insulin) (St Louis, Missouri, USA). Lantus (5B005C) was obtained from Sanofi (Paris, France) and used as positive control. Recombinant human insulin injection (H-Insulin) was obtained from Lilly. Human insulin radioimmunoassay kit, Canine C-peptide RIA kit and 3 T3-L1 mouse embryonic fibroblasts were obtained from Merck, Darmstadt, Germany.

### Test animals

Male specific pathogen free (SPF) grade Sprague Dawley (SD) rats were purchased from Beijing Veitong Lihua Co., Ltd., China. All animal experiments were in compliance with the standard operating procedures of laboratory animals specified by WuXi AppTec Institutional Animal Care and Use Committee (IACUC). Eight weeks old SD rats were kept at 20–24 °C room temperature, with humidity of 40–70% and on a 12:12 h long light-dark cycle.

Beagle dogs were purchased from Beijing Masi Animal Center. The strains, animal administration and blood collection operations of the experimental animals used in this study was approved by IACUC of Tianjin Pharmaceutical Research Institute New Drug Evaluation Co., Ltd. The dogs were kept separately in the cage with length×width×height of 90 cm × 100 cm × 90 cm. The temperature was set at 16 °C to 26 °C (measured as 18.65 °C to 27.07 °C), humidity 40 to 70% (measured as 28.50 to 94.79%), ventilation frequency of no less than 8 new winds / hour, and on a 12:12 h long light-dark cycle.

After the experiments done, the animals were euthanized with carbon dioxide, as follows:

- Place an animal in the chamber. Turn on CO2 at a low flow rate of less than 1 l per minute.
- Place tubing through hole in the lid and leave CO2 running for 2 min or until the animal stops breathing or all movements stop.
- Turn off CO2. Leave the lid on to expose the animals to CO2 gas for another 5 min. Check heartbeat and respiration to verify death.
- Perform cervical dislocation, then place the animals in the bags for disposal.
- Remove animals, appropriately label and place in the carcass freezer.

### Modeling of STZ rats

SD rats were fasted overnight for 16 h and were injected with streptozotocin 65 mg/kg intraperitoneally. After 1 h, the animals resumed eating. Fasting blood glucose was measured 5 days after the injection. The rats with fasting blood glucose greater than 16.7 mmol/L were selected as the successful type 1 diabetes model.

### Effect of single administration on blood glucose in normal rats and diabetic rats

Grouping: a total of 6 groups included 5 groups of diabetic rats and a normal control group. Normal rats were randomly assigned into 5 groups, namely vehicle group, positive control group (Lantus 1.5 IU/Kg), Lisargine of 0.5 IU/Kg, 1.5 IU/Kg, and 4.5 IU/Kg groups, 10 for each. Diabetic rats included vehicle control group, positive control group (Lantus 5 IU/Kg), Lisargine of 2.5 IU/Kg, 5 IU/Kg, and 10 IU/Kg groups, 10 for each. The subcutaneous administration volume was 2 ml/Kg. After dosing, the animals were fasted. Tail blood of normal rats were collected at 1, 2, 4, 6, 8, and 10 h after dosing, and diabetic rats at 1, 2, 4, 6, 8, 10, 12, and 24 h. Blood glucose was measured using a glucometer, according to the instructions of the kit.

### Effect of multiple administrations on blood glucose in diabetic rats

Grouping: a total of 6 groups included 5 groups of diabetic rats and a normal control group. Diabetic rats included vehicle control group, positive control group (Lantus 5 IU/Kg), Lisargine of 2.5 IU/Kg, 5 IU/Kg, and 10 IU/Kg groups, 10 for each. Before administration, there is no fasting. Insulin was injected subcutaneously for 7 consecutive days, and the same volume of vehicle was used in control group. After the administration on the 7th day, the animals were fasted for a whole course. Tail blood of STZ rats were collected at 1, 2, 4, 6, 8, 10, 12, and 24 h after dosing. Blood glucose was measured using a glucometer, according to the manufacturer’s instructions.

### Receptor affinity test of type a insulin receptor, type B insulin receptor and insulin-like growth factor IGR-1 receptor

The 100 μL reaction system was used, including 58 μL cell membrane, 2 μL test sample and 40 μL isotope, in duplicates. All test samples were diluted 10-points 3-fold, starting at a concentration of 300 nM. The cell membrane solution and [^125^I] labeled insulin with the detection buffer were prepared, the final concentration of the cell membrane was 1–3 μg, and the final concentration of [^125^I] labeled insulin was 100 pM. Along with a high-signal control well and a low-signal control well, the 96-well plate was sealed, and then incubated at room temperature for 1 h. Meaning while, soak the GF/C filter plate with 0.3% PEI. After the incubation, collect the cells from GF/C filter plate with a cell collector, wash four times with the plate washing buffer, and dry in an oven at 50 °C for 1 h. The bottom of the dried GF/C filter plate was sealed, and 50 μL of scintillation liquid was added to each well. It was sealed and read with Microbeta.

### Receptor autophosphorylation test of type a insulin receptor, type B insulin receptor

The cells were cultured in Corning 384-well plate with 50 μL/well and placed in a 5% CO_2_ incubator at 37 °C overnight. A high-signal control well and a low-signal control well are provided. On the second day, all samples were diluted in a 10-point 3-fold gradient with F12 medium containing 0.1% bovine serum albumin (BSA) with an initial concentration of 1 μM. The cell plate was removed from the incubator, the medium was shaken off with a plate washer, 25 μL of the gradient diluted samples were added and incubated at room temperature for 5 min. The medium was shaken off and 25 μL of 1 × cell lysis buffer was added. The lysate obtained in the previous step 10 times with 1× cell lysis buffer was diluted, 10 μL of lysate was added to Optiplate 384-well plate. Add 5 μl of the Acceptor mixture in the phosphorylation detection kit, seal the plate with foil, and incubate at room temperature for 1 h. Add 5 μL of the Donor mixture in the phosphorylation detection kit (protected from light) and incubate at room temperature for 1 h. Envision was used to read the plate.

### Adipocyte glucose uptake test

The 3 T3-L1 mouse embryonic fibroblasts were cultured for induction of adipogenesis, and lysate of 3 T3-L1 adipocytes were plated for glucose uptake. Krebs-Ringer-Phosphate-HEPES (KRPH) buffer was used to prepare 10-point 3-fold gradient diluted samples. After starvation, the adipocytes were rinsed with KRPH buffer. Ninety μL of gradient-diluted samples of different concentrations were added to each well with high- and low-signal control wells and incubated in a CO_2_ incubator for 30 min. Add 10 μL/well of KRPH buffer containing 0.25 μCi1- [3H] -deoxyglucose and 50 μM 2-deoxyglucose and incubate in a CO_2_ incubator for 20 min. Wash the cells with DPBS containing 10 mM glucose at 4 °C. Lysate fat cells with sodium hydroxide solution, transfer to scintillation tube, add scintillation fluid, and count with TriCarb.

### PK test in beagle dogs

The PK test of the double-dose test preparation for subcutaneous injection of animals was 4 IU and 8 IU per dog, respectively. The subcutaneous injection dose of the reference control Lantus injection was 4 IU per dog. Animals were fasted after dinner at about 21:00 on the night before the experiment administration (the second meal was 8 h after administration). At about 9:00 the next morning, the test drug or reference preparation were administrated separately, and about 2.5 mL blood from 0 h before and 1, 2, 3, 4, 5, 6, 8, 12, 16, 24 h after administration were taken, heparin anticoagulation was centrifuged at 3000 rpm for 10 min, plasma was separated, and it was immediately frozen at − 80 °C to be tested. Using high-sensitivity radioimmunoassay, the total insulin and canine C-peptide concentrations of plasma samples collected at various time points were measured. The background deduction formula of the exogenous insulin drug concentration was: Exogenous insulin concentration at time T = total insulin concentration at time T-(Insulin concentration at time 0 × C peptide concentration at time T/C peptide concentration at time 0). Human insulin and canine C peptide radioimmunoassay kits were all products of Merck, USA.

DAS2.0 PK calculation program software was used for PK parameter calculation and bioequivalence analysis. While blood was collected at each time point, a drop of whole blood was taken immediately and the blood glucose value of the animal at each time point was measured with a Roche blood glucose meter and test paper.

### Safety assessment

Hypoglycemia, death, bleeding, and other adverse events were recorded.

### Statistical analysis

Statistical analysis of the data was performed by Graphpad Prism 6.0 software, using *t*-test (two-tailed, equal variance), one-way or two-way analysis of variance (ANOVA) comparison method, with *P* < 0.05 as the criterion of significant difference. The main PK parameters (AUC (0-∞) and Cmax) were analyzed by ANOVA two-way, one-sided *t*-test and 90% confidence interval analysis, and the Tmax was tested by nonparametric test (paired Wilcoxon test).

## Results

### The effect of single administration on blood glucose in normal rats and diabetic rats and the effect of multiple administrations on blood glucose in diabetic rats.

**Fig. 1:**
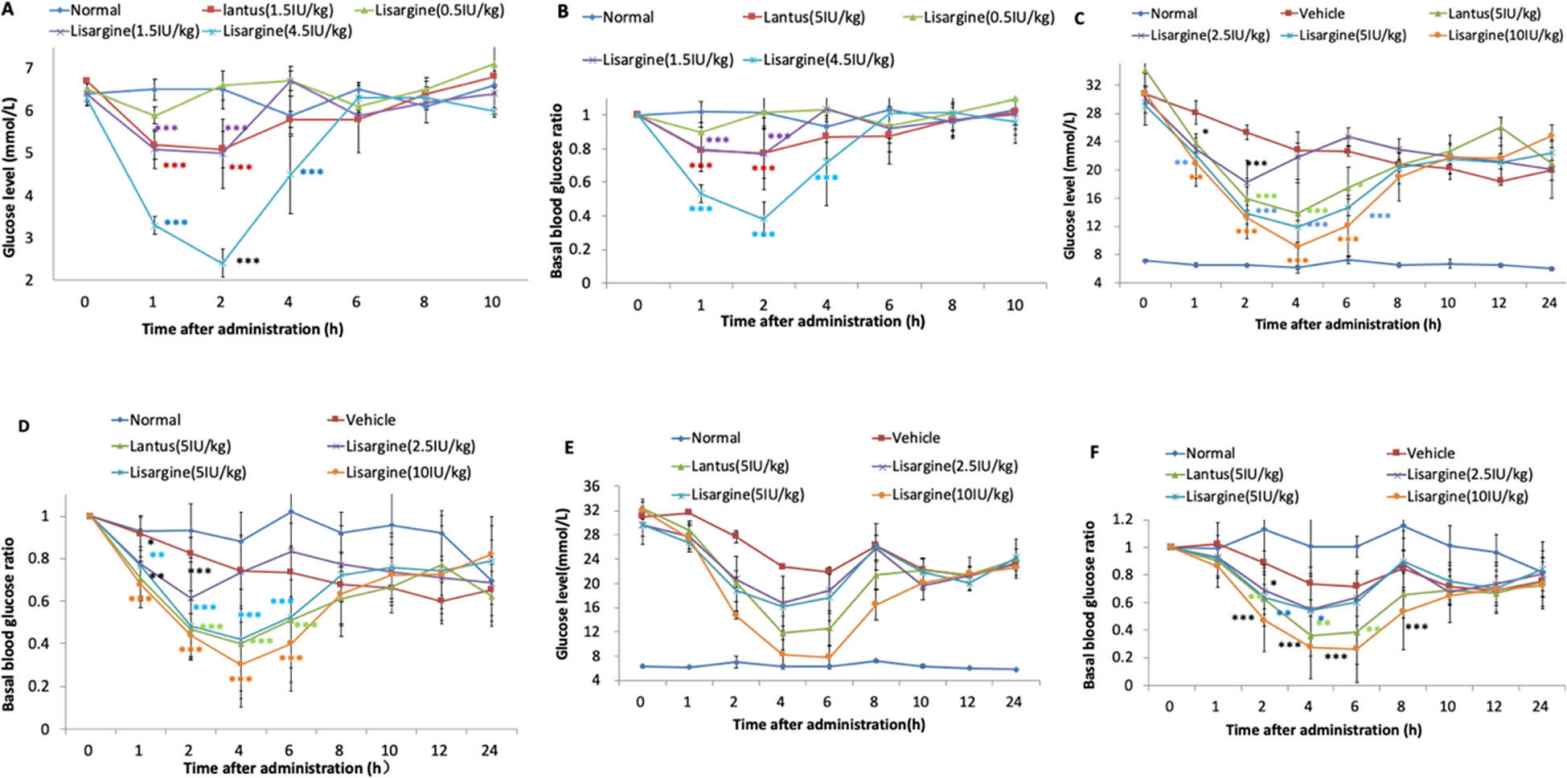
Blood glucose change curve (**a**) and basal blood glucose change curve (**b**) of SD rats after administration. The blood glucose change curve (**c**) and basal blood glucose change curve (**d**) of diabetic rats after administration. The blood glucose change curve (**e**) and basal blood glucose change curve (**f**) of diabetic rats after multiple administrations. * *P* < 0.05, ** *P* < 0.001, ** P < 0.001, compared with vehicle group

After a single subcutaneous administration of 1.5 IU/kg Lantus, the blood glucose significantly reduced at 1 h and 2 h, most significant at 2 h. With 0.5 IU/kg Lisargine, there was no change of blood glucose. With 1.5 IU/kg Lisargine, there were significant hypoglycemic effects at 1 and 2 h, most significant at 2 h, similar as same dose of Lantus. The hypoglycemic effects of the 4.5 IU/kg dose group were more significant **(**Fig. 1a and b**)**.

The trend of fluctuations in glucose level was similar between Lantus and Lisargine of same dose. The blood glucose levels of Lantus group were significantly different from that of vehicle control group at 1, 2, 4, and 6 h after administration, and the hypoglycemic effect at 4 h was the most significant at diabetic rats. For 5 IU/kg and 10 IU/kg Lisargine groups, the blood glucose levels at 1, 2, 4, and 6 h were significantly lower than those in vehicle control group, and the hypoglycemic effect was most significant at 4 h for diabetic rats **(**Fig. 1c and d**)**. The blood glucose level of the Lantus group was significantly different from that of vehicle control group at 2, 4, and 6 h after multiple administrations. For 2.5 IU/kg and 5 IU/kg Lisargine groups, the blood glucose levels at the 2 and 4 h were significantly lower than that of vehicle control group, and the hypoglycemic effect was most significant at 4 h (Fig. 1e and f**)**.

### Receptor affinity test of type a receptor, type B insulin receptor and insulin-like growth factor IGR-1 receptor

S-Insulin was used as a standard control. All samples were repeated 3 times. The IC_50_ was repeated 3 times, and the data were consistent, confirming that the experimental system was reliable and repeatable. For the binding ability to type A insulin receptor and type B insulin receptor, the recombinant insulin Lisargine injection (Lisargine) produced by Tianmai, the recombinant human-derived insulin produced by Sigma (S-Insulin, a regular insulin), Human insulin injection (H-Insulin, a regular insulin) and the Lantus obtained by Sanofi (Lantus) were similar, with no statistical difference (Tables 1, 2, and 3). Insulin growth factor-1 (IGF-1) receptor binding was lower in both Lantus and Lisargine, compared with S-Insulin (*P* < 0.05).

**Table 1:** Summary of experimental data of type A insulin receptor binding

Samples	*N* = 1	*N* = 2	*N* = 3	Mean ± SEM	*P* value	
	IC_50_ (nM)	IC_50_ (nM)	IC_50_ (nM)		vs. S-Insulin	Lantus vs. Lisargine
S-Insulin	0.93	0.82	0.79	0.82 ± 0.02	/	/
Lantus	0.82	0.65	0.30	0.50 ± 0.09	0.180	/
H-Insulin	0.76	0.51	0.49	0.54 ± 0.04	0.058	/
Lisargine	0.69	0.74	0.32	0.52 ± 0.09	0.135	0.990

*IC50* half maximal inhibitory concentration, *SEM* standard error of mean. *S-Insulin* Sigma Insulin, *H-Insulin* Human Insulin injection

**Table 2:** Summary of experimental data of type B insulin receptor binding

Samples	*N* = 1	*N* = 2	*N* = 3	Mean ± SEM	*P* value	
	IC_50_ (nM)	IC_50_ (nM)	IC_50_ (nM)		vs S-Insulin	Lantus Vs. Lisargine
S-Insulin	0.98	0.83	0.42	0.58 ± 0.09	/	/
Lantus	0.58	0.50	0.42	0.45 ± 0.02	0.228	/
H-Insulin	0.69	0.51	0.45	0.51 ± 0.04	0.342	/
Lisargine	1.04	0.76	0.55	0.70 ± 0.08	0.861	0.129

*IC50* half maximal inhibitory concentration, *SEM* standard error of mean. *S-Insulin* Sigma Insulin, *H-Insulin* Human Insulin injection

**Table 3:** Summary of experimental data of insulin growth factor-1 (IGF-1) receptor binding

Samples	*N* = 1	*N* = 2	*N* = 3	Mean ± SEM	*P* value	
	IC_50_ (nM)	IC_50_ (nM)	IC_50_ (nM)		Vs. S-Insulin	Lantus vs. Lisargine
S-Insulin	132.6	70.5	66.0	78.6 ± 10.8	/	/
Lantus	12.6	12.3	13.4	12.9 ± 0.2	0.023	/
H-Insulin	49.2	52.8	39.2	45.4 ± 2.8	0.123	/
Lisargine	12.5	16.6	10.0	12.6 ± 1.3	0.024	0.897

*IC50* half maximal inhibitory concentration, *SEM* standard error of mean. *S-Insulin* Sigma Insulin, *H-Insulin* Human Insulin injection

### Receptor autophosphorylation test of type a insulin receptor, type B insulin receptor

The autophosphorylation ability for insulin receptors of type A and B of Lisargine was similar to the recombinant human-derived insulin (H-Insulin) and Lantus, with no statistical difference **(**Table 4 and 5**)**.

**Table 4:** Summary of experimental data of autophosphorylation of type A insulin receptor

Samples	*N* = 1	*N* = 2	*N* = 3	Mean ± SEM	*P* value	
	EC_50_ (nM)	EC_50_ (nM)	EC_50_ (nM)		vs. S-Insulin	Lantus vs. Lisargine
S-Insulin	17.5	23.7	12.4	17.0 ± 2.3	/	/
Lantus	12.8	17.2	18.7	17.2 ± 0.9	0.681	/
H-Insulin	15.7	15.8	20.4	18.1 ± 1.0	0.884	/
Lisargine	18.2	11.6	31.3	22.6 ± 4.0	0.725	0.531

*IC50* half maximal inhibitory concentration, *SEM* standard error of mean. *S-Insulin* Sigma Insulin, *H-Insulin* Human Insulin injection

**Table 5:** Summary of experimental data on autophosphorylation of type B insulin receptor

Samples	*N* = 1	*N* = 2	*N* = 3	Mean ± SEM	*P* value	
	EC_50_ (nM)	EC_50_ (nM)	EC_50_ (nM)		vs S-Insulin	Lantus vs. Lisargine
S-Insulin	24.6	8.5	31.5	22.7 ± 4.6	/	/
Lantus	17.6	6.6	21.8	16.0 ± 3.1	0.489	/
H-Insulin	28.3	9.8	17.8	16.9 ± 2.8	0.752	/
Lisargine	29.0	7.9	20.1	17.5 ± 3.4	0.796	0.653

*IC50* half maximal inhibitory concentration, *SEM* standard error of mean. *S-Insulin* Sigma Insulin, *H-Insulin* Human Insulin injectionn

### Adipocyte glucose uptake test

With Lisargine, adipocytes’ glucose uptake was similar to that with H-Insulin, S-Insulin and Lantus **(**Table 6**)**.

**Table 6:** Summary of experimental data of fat cell glucose uptake

Samples	*N* = 1	*N* = 2	*N* = 3	Mean ± SEM	*P* value	
	EC_50_ (nM)	EC_50_ (nM)	EC_50_ (nM)		vs S-Insulin	Lantus vs. Lisargine
S-Insulin	2.5	5.3	3.0	3.7 ± 0.5	/	/
Lantus	1.6	3.8	2.9	3.0 ± 0.3	0.506	/
H-Insulin	0.9	1.7	2.2	1.8 ± 0.2	0.102	/
Lisargine	3.5	8.5	4.5	5.7 ± 0.9	0.333	0.178

*IC50* half maximal inhibitory concentration, *SEM* standard error of mean. *S-Insulin* Sigma Insulin, *H-Insulin* Human Insulin injection

### PK test in beagle dogs

**Fig. 2:**
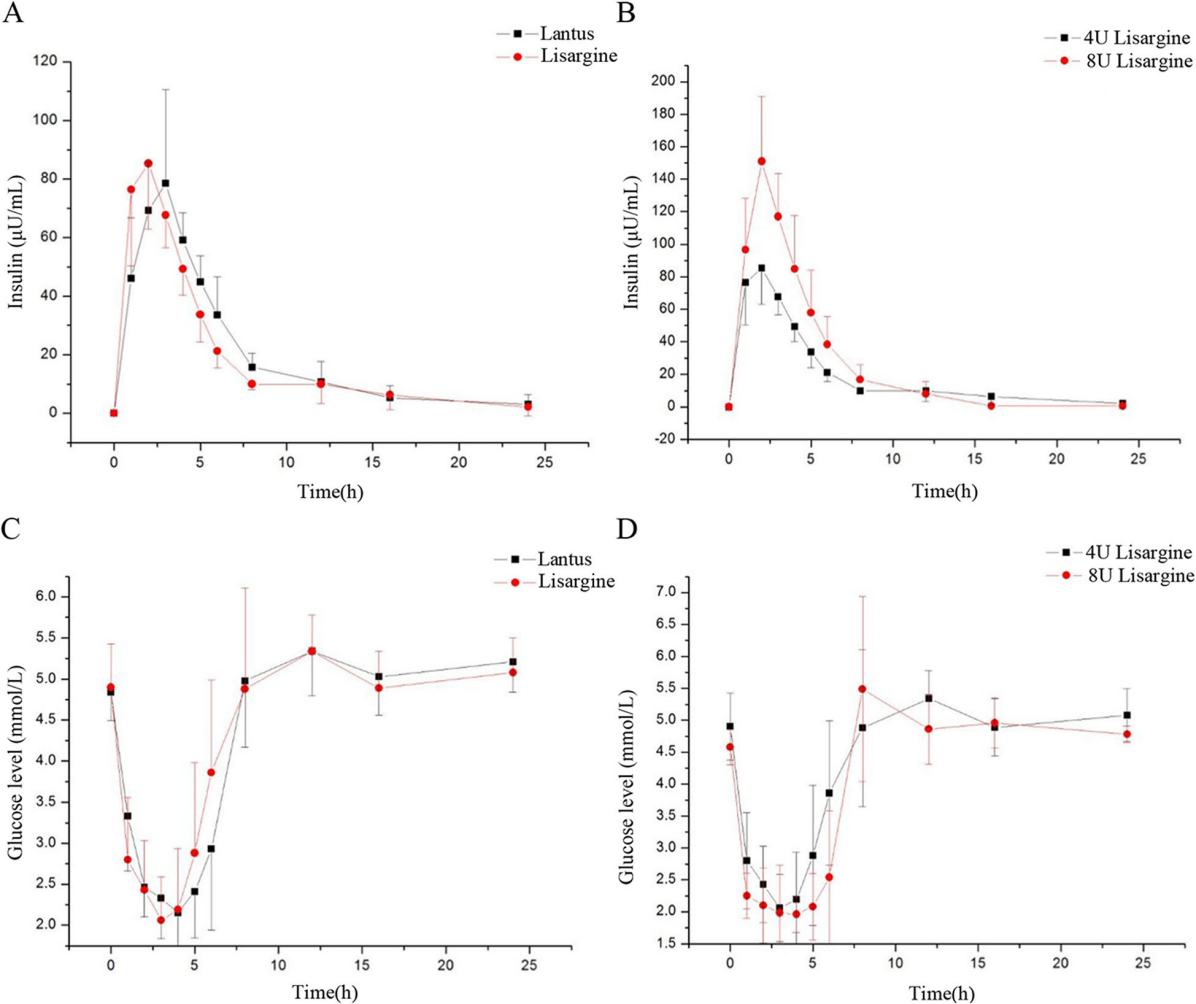
Curve of average exogenous insulin administration time after cross-SC Lisargine and Lantus (4 U) in 8 Beagle dogs (**a**). Curve of average exogenous insulin administration time after SC Lisargine (4 U and 8 U) in 8 Beagle dogs (**b**). The average blood glucose concentration-time curve after cross-SC Lisargine and Lantus (4 U) in 8 Beagle dogs (**c**). The average blood glucose concentration-time curve after SC Lisargine (4 U and 8 U) in 8 Beagle dogs (**d**)

For PK test, subcutaneous injection of 4 IU and 8 IU Lisargine per dog was used and the dose of 4 IU Lisargine was used as reference (control). The results showed that after the high and low doses of Lisargine, T1/2 of the high-dose group was significantly shorter than that of the low-dose group with Lisargine. The Tmax of the two groups was close. There was no statistical difference for PK parameters between Lisargine and Lantus, 4 U/dog, except a slight right shift of Lantus for peak time. The relative bioavailability of the test drug Lisargine to the control drug Lantus was 94.5%. It showed that the PK behavior of the two drugs on healthy Beagle dogs were similar **(**Fig. 2**)**. For area under curve (AUC), as shown in Table 7, there was no difference for 4 IU of Lantus and Lisargine, but it is higher with Lisargine 8 IU (*P* < 0.05 compared with Lisargine 4 IU or Lantus 4 IU).

**Table 7:** Area Under Curve (AUC) for Lisargine and Lantus within 24 h

	AUC total	P
Lantus 4 IU	498	0.37 *
Lisargine 4 IU	461	0.02 **
Lisargine 8 IU	643	0.005 ***

*, Lantus 4 IU vs. Lisargine 4 IU. **, Lisargine 4 IU vs. Lisargine 8 IU; ***, Lisargine 8 IU vs. Lantus 4 IU

**Safety**

No adverse event was reported in any group.

## Discussion

Diabetes is a chronic disease [11], a major harm to human health in the twenty-first century [12, 13]. Blood glucose control is the key for diabetes treatment and prevention of its complications [14]. Exogenous insulin is mainly used in patients with type 1 diabetes and type 2 diabetes poorly controlled with diet and oral anti-diabetic drug (OAD). Medium-acting insulin have the disadvantages of high variability, obvious peak effect, shorter duration, and high risk of hypoglycemia at night [15, 16]. Long-acting insulin is needed so that patients can control their blood glucose with single injection only once a day [17].

One common long-acting insulin is glargine, Lantus. After being injected, its solubility reduces and it forms into small precipitate. These particles slowly dissolve into monomers and release into blood. The 24 h insulin level is similar to the basal insulin in healthy people. It lowers blood sugar smoothly, steadily and for longer durations.

Lisargine is a new type of insulin lysine injection. In this study, through comparing PD and PK of Lisargine and Lantus, we explored their bioequivalence, with the hope that Lisargine would be an effective addition to Lantus.

Our study suggested that with the 1.5 IU/kg dose of Lisargine, there was significant hypoglycemic effect after 1 and 2 h of administration, and the hypoglycemic effect was most significant at 2 h, which was similar to the same dose of Lantus. The blood glucose levels of diabetic rats were significantly reduced by Lisargine 5 IU/Kg and 10 IU/Kg at 2, 4, and 6 h, and by Lisargine 2.5 IU/Kg at 1 h and 2 h. With 5 IU/Kg, Lisargine reduced blood glucose similar to reference drug Lantus.

The binding abilities of Lisargine, S-Insulin, H-Insulin, and Lantus to type A insulin receptor and type B insulin receptor were similar. But Lantus and Lisargine have lower binding effect to IGF-1, compared with S-Insulin. Possibly, their action may not mainly through activating IGF-1. There was no difference for receptor autophosphorylation of test-type A insulin receptor and type B insulin receptor between Lisargine and reference drug (H-Insulin and Lantus). Glucose uptake of adipocytes was similar for all. This supported that Lisargine was as effective as Lantus.

In comparing PK and PD of glargine and Lantus, smiliar resutls were found across studies [18]. The primary PK parameters (area under curve from 0 to 24 h [AUC_0–24_]) and PD parameters were not statistically different between Lisargine and Lantus. No safety concern was noted with either drug. PK and PD parameters for glargine and Luntas were comparable following single doses [19]. Yet, in this reference study, 0.3 and 0.6 U/kg Lantus were effective while Lisargine 0.5 U/kg was not effective in our study. It may be due to the fact that Lisargine has a lysine in both chains while Lantus has a glycine.

The Tmax of Lisargine showed no statistical difference from Lantus. The PK parameters of Lisargine group were comparable with Lantus at the same dose. Lantus did have a slight right shift of the peak time. As lysine is a key amino acid for Lisargine while glycine for Lantus, this may be because lysine takes action faster than glycine. Our results showed that the PK behavior of the two drugs on healthy Beagle dogs was similar and the relative bioavailability of Lisargine to Lantus was 94.5%. Another study found the bioequivalence of Glargine to Lantus with 90% confidence interval within 0.80–1.25 [20], indicating PK equivalence between the biosimilar and reference Lantus [21]. This study was limited to animals. Further study will focus on healthy volunteers and diabetic patients to verify the bioequivalence of Lisargine with Lantus and its safety.

## Conclusions

In a conclusion, the hypoglycemic effects and PD of Lisargine in diabetic rats were comparable with those of Lantus, and PK parameters of Lisargine were similar with that of Lantus in Beagle dogs.

## Data Availability

Not applicable.

## Abbreviations

PD: Pharmacodynamic
PK: Pharmacokinetic
IACUC: Institutional Animal Care and Use Committee
